# Topotecan and Ginkgolic Acid Inhibit the Expression and Transport Activity of Human Organic Anion Transporter 3 by Suppressing SUMOylation of the Transporter

**DOI:** 10.3390/pharmaceutics16050638

**Published:** 2024-05-09

**Authors:** Zhou Yu, Guofeng You

**Affiliations:** Department of Pharmaceutics, Rutgers, The State University of New Jersey, 160 Frelinghuysen Road, Piscataway, NJ 08854, USA

**Keywords:** drug transporter, organic anion transporter 3, regulation, topotecan, SUMOylation, post-translational modification

## Abstract

Organic anion transporter 3 (OAT3), expressed at the basolateral membrane of kidney proximal tubule cells, facilitates the elimination of numerous metabolites, environmental toxins, and clinically important drugs. An earlier investigation from our laboratory revealed that OAT3 expression and transport activity can be upregulated by SUMOylation, a post-translational modification that covalently conjugates SUMO molecules to substrate proteins. Topotecan is a semi-synthetic derivative of the herbal extract camptothecin, approved by the FDA to treat several types of cancer. Ginkgolic acid (GA) is one of the major components in the extract of *Ginkgo biloba* leaves that has long been used in food supplements for preventing dementia, high blood pressure, and supporting stroke recovery. Both topotecan and GA have been shown to affect protein SUMOylation. In the current study, we tested our hypothesis that topotecan and GA may regulate OAT3 SUMOylation, expression, and transport function. Our data show that the treatment of OAT3-expressing cells with topotecan or GA significantly decreases the SUMOylation of OAT3 by 50% and 75%, respectively. The same treatment also led to substantial reductions in OAT3 expression and the OAT3-mediated transport of estrone sulfate, a prototypical substrate. Such reductions in cell surface expression of OAT3 correlated well with an increased rate of OAT3 degradation. Mechanistically, we discovered that topotecan enhanced the association between OAT3 and the SUMO-specific protease SENP2, a deSUMOylation enzyme, which contributed to the significant decrease in OAT3 SUMOylation. In conclusion, this study unveiled a novel role of topotecan and GA in inhibiting OAT3 expression and transport activity and accelerating OAT3 degradation by suppressing OAT3 SUMOylation. During comorbidity therapies, the use of topotecan or *Ginkgo biloba* extract could potentially decrease the transport activity of OAT3 in the kidneys, which will in turn affect the therapeutic efficacy and toxicity of many other drugs that are substrates for the transporter.

## 1. Introduction

The organic anion transporter (OAT) family, consisting of more than 10 members, belongs to the solute carrier superfamily of membrane proteins. OATs move organic anions in and out of cell membranes. OAT1 and OAT3 are mainly expressed in the kidneys where they are the major determinants in renal elimination of numerous endogenous and exogenous organic solutes such as nutrients, metabolites, toxins, and drugs [1,2,3,4,5,6]. Consequently, both transporters are required by FDA to be studied during drug development [5,7,8,9,10]. Therefore, the elucidation of the mechanisms underlying the regulation of these transporters will provide insights into our understanding of the roles of these transporters in renal physiology, injury, diseases, and their applications to drug therapy.

As a membrane protein, the transport activity of OATs critically relies on its expression level at the cell surface [1,2,3,5]. Our lab previously revealed that OATs are constitutively internalized from the plasma membrane to intracellular endosomes. Once in the endosomes, the internalized OATs can either recycle back to the plasma membrane or target the proteasomes for degradation [11,12]. The cell surface expression and, therefore, the transport activity of OATs can be regulated by altering any steps of their trafficking process: meaning altering the rates of internalization, recycling, and/or degradation [2,3]. Our lab demonstrated that OAT activity can be upregulated by SUMOylation through reducing the rates of OAT internalization and degradation [13,14,15,16]. SUMOylation is a post-translational modification, in which a SUMO molecule is attached to the target protein to regulate its expression and functions [17,18,19,20]. SUMOylation involves a three-step enzyme cascade to conjugate SUMO molecules to lysine residues on substrate proteins covalently. The SUMOylation enzymes include SUMO-activating enzyme E1 (SAE1/SAE2), SUMO-conjugating enzyme E2 (Ubc9), and SUMO ligase E3 (PIAS family members) [19]. Additionally, members from the specific SUMO isopeptidase family, SENPs, facilitate the reversal of this modification, deSUMOylation [19,21,22,23,24]. All these enzymes can be the potential targets for the regulation of OAT activity.

Topotecan is a semi-synthetic derivative of camptothecin, an herbal extract from the Chinese yew tree, *Camptotheca acuminata*. It has been approved by the US Food and Drug Administration to treat several types of solid tumors such as cervical, ovarian, and small cell lung cancer [25,26,27]. Its known mechanism of action is to inhibit DNA topoisomerase I, through which it exerts its cytotoxic effects on cancer cells [28,29,30,31,32]. Other functions of topotecan have also been reported, such as reducing protein SUMOylation levels in human glioblastoma multiforme; therefore, inducing significant changes in cell cycle and cell metabolism [28,29]. Ginkgolic acid (GA) is one of the major components in the extract of *Ginkgo biloba* leaves that has long been used in traditional Chinese medicine and as a food supplement for preventing dementia and high blood pressure, and supporting stroke recovery [30,33,34]. Recently, GA has been identified as a small molecule inhibitor for protein SUMOylation [33,35]. For instance, in rat kidney RPTC cells, GA treatment suppressed protein SUMOylation and increased cell apoptosis during cisplatin incubation [35]. In addition, both topotecan and GA have been reported to regulate kidney functions in vivo [31,36,37]. However, their effects on OAT3 expression and transport activity have never been investigated. In the current study, we examined the effects of topotecan and GA on OAT3 SUMOylation, protein expression, and transport function.

## 2. Materials and Methods

### 2.1. Materials

[^3^H]-labeled estrone sulfate (ES) was purchased from PerkinElmer (Waltham, MA, USA). Membrane-impermeable biotinylation reagent Sulfo-NHS-SS-biotin, streptavidin agarose resin, protein G agarose resin, an anti-mouse HRP-linked antibody, and an anti-rabbit HRP-linked antibody were purchased from Thermofisher Scientific (Waltham, MA, USA). A mouse anti-myc antibody (clone 9E10) was bought from Roche (Indianapolis, IN, USA). Rabbit anti-SUMO2/3, anti-Ubc9, and anti-SENP2 antibodies were purchased from Abcam (Cambridge, MA, USA). Topotecan HCL, ginkgolic acid (GA), and 2-D08 were obtained from Selleck Chemicals (Houston, TX, USA). All other reagents were purchased from Sigma-Aldrich (St. Louis, MO, USA).

### 2.2. Cell Culture and Transfection

Parental monkey kidney COS-7 cells and human embryonic kidney HEK293 cells were purchased from ATCC (Manassas, VA, USA). Parental COS-7 and HEK293 cells were grown in regular DMEM medium (Invitrogen, Carlsbad, CA, USA) with 10% fetal bovine serum (FBS) (Thermofisher Scientific, Waltham, MA, USA) under a temperature of 37 °C and 5% CO_2_ in a standard cell culture incubator (Thermofisher Scientific, Waltham, MA, USA). COS-7 cells and HEK293 cells stably expressing human OAT3 were previously established in our lab as described [38]. These stable OAT3 cells were grown in regular DMEM medium with 10% FBS and 0.5 mg/mL G418 antibiotic (Invitrogen, Carlsbad, CA, USA). DNA transfection was conducted using the Lipofectamine 3000 reagent kit (Invitrogen, Carlsbad, CA, USA) according to the standard protocol from Invitrogen. The transfected cells were grown in a cell culture incubator for 48 h before the following experiments were performed.

### 2.3. Transport Activity Measurement (Uptake Assay)

The uptake assay was performed in COS-7 and HEK293 cells stably expressing OAT3 according to the established protocol in our lab [11,13,14,39]. In brief, cells were plated in 48-well culture plates 24 h before the uptake assay. After removing culture media, an uptake solution consisting of phosphate-buffered saline (PBS) with 0.3 μM [^3^H]-estrone sulfate (ES) was added to start the uptake assay at room temperature. The cellular uptake process was terminated by aspirating the uptake solution and washing cells with cold PBS. The cells were then lysed with 0.2 N NaOH and neutralized with 0.2 N HCl. The cell lysates were measured by liquid scintillation counting using a Beckman LS6500 multi-purpose scintillation counter (Beckman Coulter, Brea, CA, USA). Transport activity values were plotted as a percentage of those in control cells.

### 2.4. Biotinylation Assay

The assessment of OAT3 protein expression at the cell membrane was performed using a biotin-labeling strategy established in our lab [11,13,14]. In brief, cells were plated in culture dishes 24 h before the assay. Membrane-impermeable reagent Sulfo-NHS-SS-biotin (0.5 mg/mL in PBS pH 8.0) was added to label cell surface proteins. After the labeling step, the unreacted Sulfo-NHS-SS-biotin was quenched with 100 mM glycine in PBS and the cells were washed multiple times with PBS. After 1 h cell lysis at 4 °C with end-to-end rotation, the cell lysates were centrifugated at 15,000× *g* at 4 °C to remove cell debris. The supernatants after centrifugation were added to streptavidin resin to separate the biotin-labeled proteins from the cell surface. OAT3 at the cell surface was identified via SDS-PAGE and western blotting. E-cadherin was measured as a marker for cell surface proteins. OAT3 expression in whole-cell lysates was directly measured by SDS-PAGE and western blotting. GAPDH was analyzed as a total protein marker.

### 2.5. Degradation Assay

The assessment of the degradation rate of OAT3 was conducted following the protocol previously established in our lab [13,14]. In brief, OAT3-expressing COS-7 cells were first labeled with 0.5 mg/mL Sulfo-NHS-SS-biotin in PBS at 4 °C. After the quenching of the unreacted NHS-SS-biotin with glycine solution, the labeled cells were put back in regular DMEM medium containing topotecan or GA at 37 °C in a cell culture incubator. After specific time points, the cells were washed, lysed, and centrifuged at 15,000× *g* at 4 °C. The cleared cell lysates were added to streptavidin resin to separate biotin-labeled proteins, and undegraded OAT3 from the cell surface was measured by SDS-PAGE and western blotting using an anti-myc antibody. The amount of undegraded OAT3 was plotted as a percentage of the initial amount of labeled OAT3.

### 2.6. Immunoprecipitation

The immunoprecipitation assay of OAT3 was conducted based on the standard protocol established in our lab [13,14]. In short, treated cells were lysed at 4 °C, and an equal amount of total proteins (~1000 μg) was mixed with protein G resin at 4 °C for 4 h to lower non-specific binding. In the meantime, the primary antibody was mixed with protein G resin at 4 °C to create an antibody–resin complex. Then, the pre-cleared protein samples were incubated with the antibody–resin complex at 4 °C overnight with gentle shaking. Next, the proteins captured on protein G resin were denatured and eluted with Laemmli buffer from Thermofisher Scientific (Waltham, MA, USA). The eluted samples were measured by standard SDS-PAGE and western blotting with indicated primary and secondary antibodies in each experiment.

### 2.7. Cytotoxicity Assay

We performed the cytotoxicity assessment in COS-7 cells using the CyQUANT LDH Cytotoxicity Assay purchased from Invitrogen (Waltham, MA, USA). We mostly followed the standard assay protocol provided by the manufacturer [40]. In short, after the indicated treatment on cells in 96-well cell culture plates, 50 μL of sample media was transferred to a 96-well assay plate in triplicates. Then, 50 μL of reaction mixture was added to each sample media well and incubated for 30 min, protected from light. After adding 50 μL of stop solution and gently mixing, the sample absorbance was measured at 490 nm and 680 nm (as a reference wavelength) by the Infinity M PLEX microplate reader purchased from Tecan (Männedorf, Switzerland). The percentage of cytotoxicity was calculated as below: % cytotoxicity = (sample reading − medium reading)/(maximum LDH reading − medium reading) * 100. Negative controls are the treatment medium taken from untreated wells.

### 2.8. SDS-PAGE and Western Blotting

We adopted the standard protocols for SDS-PAGE and western blotting from Bio-Rad (Hercules, CA, USA) and made some optimizations [13]. In short, denatured protein samples were run through 7.5% SDS-PAGE mini-gels in Tris-glycine running buffer from Bio-Rad (Hercules, CA, USA), then electro-transferred to PVDF membranes (Bio-Rad, Hercules, CA, USA). The membranes were blocked with 5% nonfat milk in PBST buffer (0.1% Tween-20 added in PBS), followed by incubating with primary antibodies overnight at 4 °C. After washing, the blots were covered with HRP-lined secondary antibody solution for 4 h. SuperSignal West Dura Extended Duration Substrate purchased from Thermofisher Scientific (Waltham, MA, USA) was used for signal detection. A ChemiDoc imaging system (Bio-Rad, Hercules, CA, USA) was utilized to capture the immunoreactive and non-saturated bands of target proteins. Imaging Lab 6.0 software from Bio-Rad (Hercules, CA, USA) was utilized to quantify protein bands of interest. Membrane stripping was completed by incubation with Restore stripping buffer from Thermofisher Scientific (Waltham, MA, USA) for 30 min at room temperature. The stripped membranes were washed with PBST buffer before re-probing with another antibody.

### 2.9. Data Analysis

In this study, each experiment was individually repeated at least three times, and multiple experimental repeats were included for statistical analysis. Student’s *t*-tests were used for comparisons between the two groups. One-way ANOVA with post hoc Tukey’s test was used for comparison among multiple groups. GraphPad Prism 9 software (GraphPad Software, San Diego, CA, USA) was utilized for statistical analysis. A * *p*-value of <0.05 was set as statistically significant.

## 3. Results

### 3.1. Effects of Topotecan and GA on OAT3 SUMOylation

**Figure 1:**
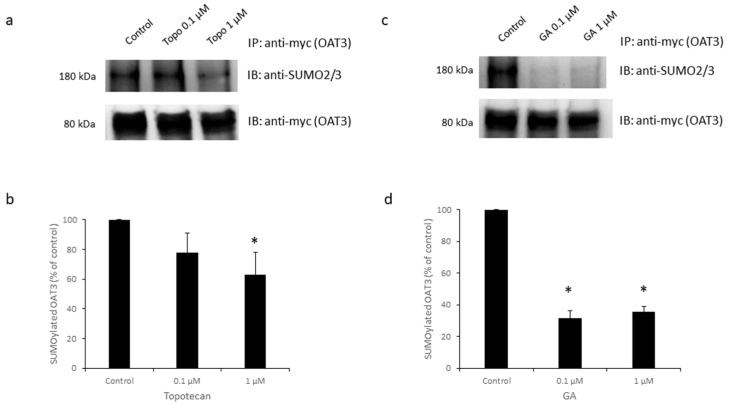
Effects of Topotecan and GA on OAT3 SUMOylation. (**a**) Top Panel: cells expressing OAT3 were treated with topotecan (0.1 or 1 µM, 12 h). OAT3 was immunoprecipitated (IP) by an anti-myc antibody and immune detected (IB) by anti-SUMO2/3 to assess the level of OAT3 SUMOylation. Bottom panel: the same blot was re-probed with an anti-myc antibody to measure the amount of pulled-down OAT3. (**b**) The densitometry of results from Figure 1a and other repeats. Density values were normalized by total OAT3 pulled down. Values are mean ± S.D. (*n* = 3). * *p* < 0.05. (**c**) Top Panel: cells expressing OAT3 were treated with GA (0.1 or 1 µM, 6 h). OAT3 was immunoprecipitated (IP) by an anti-myc antibody, and immune detected (IB) with anti-SUMO2/3. Bottom panel: The same blot was re-probed with an anti-myc antibody to measure the amount of pulled-down OAT3. (**d**) The densitometry of results from Figure 1c and other repeats. Density values were normalized by total OAT3 pulled down. Values are mean ± S.D. (*n* = 3). * *p* < 0.05.

Due to their potential role in protein SUMOylation, we first investigated whether topotecan or GA could affect the level of OAT3 SUMOylation in cultured cells. Our pilot studies showed that topotecan took a longer time than GA to generate significant effects on OAT3. Therefore, OAT3-expressing COS-7 cells were treated with topotecan for 12 h or GA for 6 h. OAT3 in treated cells was then immunoprecipitated (IP) by protein G agarose beads, followed by immunoblotting (IB) with an anti-SUMO2/3 antibody to detect SUMOylated OAT3. Our results (Figure 1) showed that 1 µM topotecan or 1 µM GA significantly decreased the SUMOylated OAT3 by 40% and 70%, respectively. The differences observed in SUMOylated OAT3 were not caused by an unequal input of total OAT3, because a similar amount of OAT3 was pulled down among all samples; these results indicate that both topotecan and GA act as inhibitors for OAT3 SUMOylation.

### 3.2. Effects of Topotecan and GA on OAT3 Transport Activity

**Figure 2:**
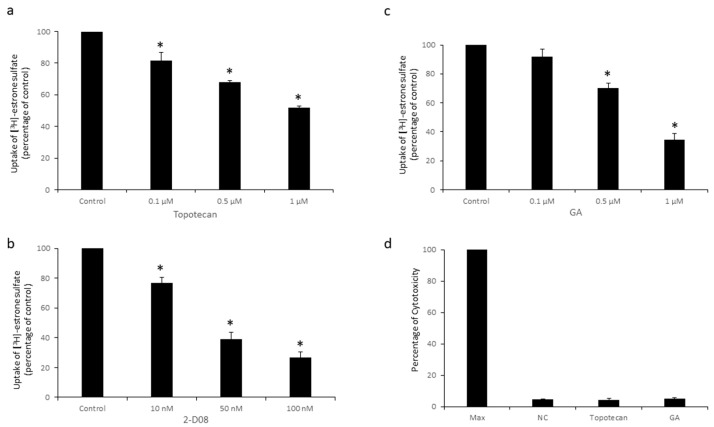
Effects of Topotecan and GA on OAT3 Transport Activity in COS-7 cells. (**a**) COS-7 cells stably expressing OAT3 were treated with multiple concentrations of topotecan (0.1–1 µM) for 12 h, followed by [^3^H]-ES uptake. Uptake activity was plotted as percentage of the values from control. The data represent uptake into OAT3-expressing cells minus uptake into parental cells. Values are mean ± S.D. (*n* = 3). * *p* < 0.05. (**b**) COS-7 cells stably expressing OAT3 were treated with various concentrations of GA (0.1–1 µM) for 6 h, followed by a [^3^H]-ES uptake assay. Values are mean ± S.D. (*n* = 3). * *p* < 0.05. (**c**) COS-7 cells expressing OAT3 were treated with various concentrations of 2-D08 (10–100 nM) for 6 h, followed by an uptake assay. Values are mean ± S.D. (*n* = 3). * *p* < 0.05. (**d**) Cytotoxicity was assessed using a cyQUANT LDH Cytotoxicity Assay after topotecan (12 h) or GA (6 h) treatment on OAT3-expressing COS-7 cells. Max, maximal LDH reading. NC, negative control. Values are mean ± S.D. (*n* = 3).

**Figure 3:**
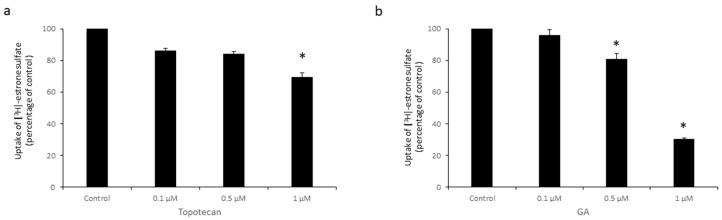
Effects of Topotecan and GA on OAT3 Transport Activity in HEK293 cells. (**a**) HEK293 cells stably expressing OAT3 were treated with multiple concentrations of topotecan (0.1–1 µM) for 12 h, followed by [^3^H]-ES uptake. Uptake activity was plotted as percentage of the uptake from control. The data represent uptake into OAT3-expressing cells minus uptake into parental cells. Values are mean ± S.D. (*n* = 3). * *p* < 0.05. (**b**) HEK293 cells expressing OAT3 were treated with various concentrations of GA (0.1–1 µM) for 6 h, followed by an uptake assay. Values are mean ± S.D. (*n* = 3). * *p* < 0.05.

Next, we studied the effects of topotecan and GA on OAT3 transport activity. OAT3-expressing COS-7 cells were treated with topotecan (Figure 2a), GA (Figure 2b), or 2-D08 (a known classic SUMOylation inhibitor [41,42], Figure 2c) individually under various doses, followed by an uptake assay to quantify the OAT3-mediated [^3^H]-estrone sulfate (ES) uptake. [^3^H]-ES was a radioisotope-labeled prototypical substrate widely utilized to measure OAT3 transport activity. Our results (Figure 2) showed that both topotecan and GA significantly reduced OAT3 transport activity in a dose-dependent manner, up to 50% and 65%, respectively. As a known SUMOylation inhibitor, 10–100 nM of 2-D08 also inhibited OAT3 transport activity markedly. The Cytotoxicity Assay indicated negligible cytotoxicity caused by topotecan and GA under the concentrations and time used (Figure 2d). Moreover, the similar effects of topotecan and GA on OAT3 transport activity were also observed in human embryonic kidney HEK293 cells (Figure 3).

### 3.3. Reversibility of the Effects of Topotecan and GA on OAT3 

**Figure 4:**
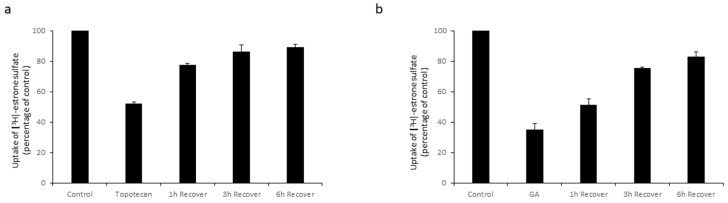
The reversibility of Topotecan and GA on OAT3 Transport Activity. (**a**) COS-7 cells expressing OAT3 were treated with topotecan (1 µM) for 12 h and allowed to recover in DMEM medium in a cell incubator for 1–6 h, followed by a [^3^H]-ES uptake assay. Uptake activity was plotted as percentage of the uptake from control. The data represent uptake into OAT3-expressing cells minus uptake into parental cells. Values are mean ± S.D. (*n* = 3). (**b**) COS-7 cells expressing OAT3 were treated with GA (1 µM) for 6 h and allowed to recover in normal DMEM medium in a cell incubator for 1–6 h, followed by a [^3^H]-ES uptake assay. Values are mean ± S.D. (*n* = 3).

Furthermore, we examined the reversibility of topotecan and GA on OAT3 transport activity. After treatment with topotecan or GA, the treated cells were washed and incubated in regular DMEM medium + 10% FBS under cell culture conditions to let them recover. After 1–6 h of recovery, OAT3 transport activity was assessed using a [^3^H]-ES uptake assay. Our results (Figure 4) showed that the inhibitory effects of topotecan and GA on OAT3 transport activity were reversible and the recovery is time dependent. The transport activity was regained more than 80% of control after 6 h recovering incubation.

### 3.4. Effects of Topotecan and GA on OAT3 Expression

**Figure 5:**
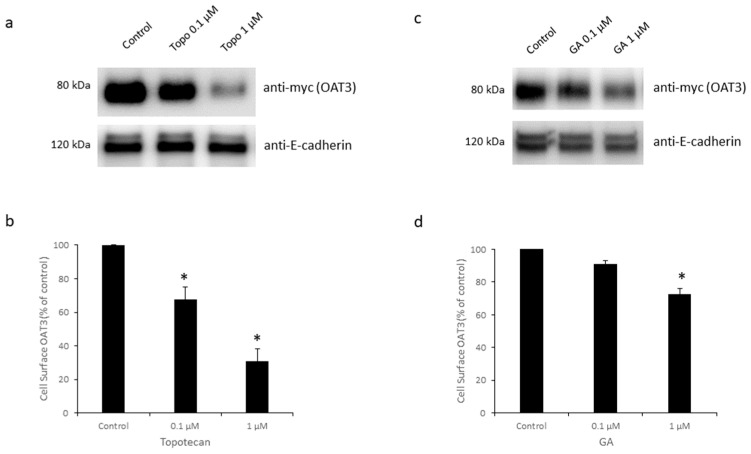
Effects of Topotecan and GA on OAT3 Expression at the Cell Surface. (**a**) Top panel: Topotecan on cell surface expression of OAT3. OAT3-expressing cells were treated with topotecan (0.1 or 1 µM, 12 h). After biotin labeling, cells were washed and lysed. Biotin-labeled proteins were captured by streptavidin resin. Immunoblotting (IB) was used to detect myc-tagged OAT3. Bottom panel: The same blot was re-probed with anti-E-cadherin. E-cadherin is a marker for cell membrane proteins. (**b**) Densitometry of Figure 5a and other repeats. Density values were normalized by E-cadherin. Values are mean ± S.D. (*n* = 3). * *p* < 0.05. (**c**) Top panel: GA on cell surface expression of OAT3. Cells expressing OAT3 were treated with GA (0.1 or 1 µM, 6 h). Bottom panel: the same blot was re-probed with anti-E-cadherin. (**d**) Densitometry of Figure 5c and other repeats. Density values were normalized by E-cadherin. Values are mean ± S.D. (*n* = 3). * *p* < 0.05.

**Figure 6:**
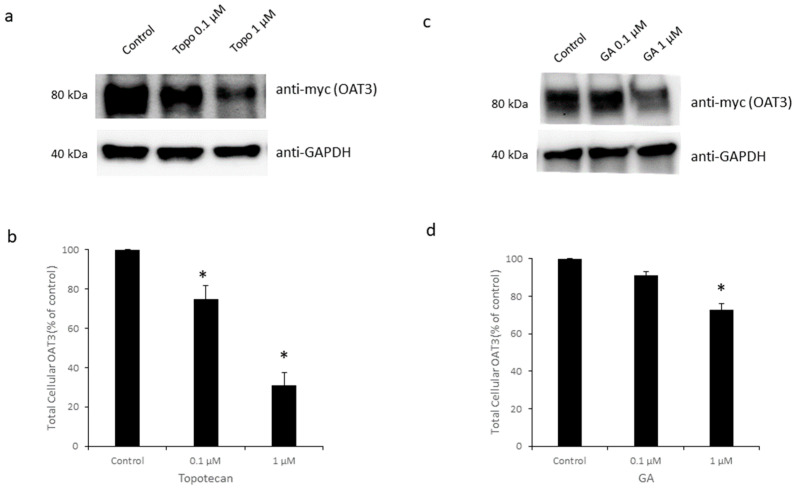
Effects of Topotecan and GA on total protein expression of OAT3. (**a**) Top panel: Topotecan on total cellular expression of OAT3. Cells expressing OAT3 were treated with topotecan (0.1 or 1 µM, 12 h). The cells were lysed and immunodetected with an anti-myc antibody. Bottom panel: the same blot was re-probed with anti-GAPDH as a total protein marker. (**b**) The densitometry of Figure 6a and other repeats. Density values were normalized to control protein GAPDH. Values are mean ± S.D. (*n* = 3). * *p* < 0.05. (**c**) Top panel: GA on total cellular expression of OAT3. Bottom panel: the same blot was re-probed with anti-GAPDH. (**d**) The densitometry of Figure 6c and other repeats. Density values were normalized to GAPDH. Values are mean ± S.D. (*n* = 3). * *p* < 0.05.

To explore the effects of topotecan and GA on OAT3 expression, we employed a biotin-labeling strategy to detect OAT3 expression at the cell surface. Sulfo-NHS-SS-biotin is a membrane impermeable reagent and only labels proteins at the cell plasma membrane. OAT3-expressing cells were treated with topotecan or GA, followed by biotin labeling, streptavidin capture, and immune detection. Our results showed that treatment with topotecan and GA significantly decreased the expression of OAT3 at the cell membrane (top panels of Figure 5a,c). We also examined the effects of topotecan and GA on OAT3 expression in whole-cell lysates. Both topotecan and GA significantly reduced OAT3 total expression (top panels of Figure 6a,c). Such alterations in OAT3 expression were not due to the variability in protein loadings, since the levels of surface protein marker E-cadherin and total protein marker GAPDH were quite similar across all groups (bottom panels of Figure 5a,c and Figure 6a,c).

### 3.5. Effects of Topotecan and GA on OAT3 Stability

**Figure 7:**
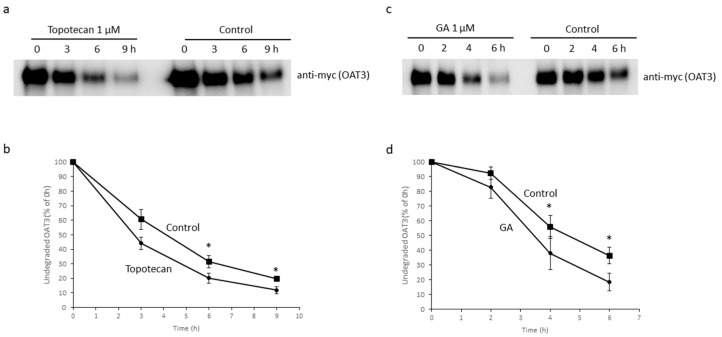
Effects of Topo and GA on OAT3 stability. (**a**) OAT3-expressing cells were firstly biotin labeled and treated with 1 µM of topotecan for 3, 6, or 9 h under normal culture conditions. The degradation rate of OAT3 was measured as described in “Materials and Methods”. (**b**) The densitometry of Figure 7a and other repeats. The undegraded OAT3 was displayed as a percentage of the amount of initial OAT3. Values are mean ± S.D. (*n* = 3). * *p* < 0.05. (**c**) OAT3-expressing cells were biotin labeled and treated with 1 µM of GA for 2, 4, or 6 h under normal culture conditions. The degradation rate of OAT3 was then measured. (**d**) The densitometry of Figure 7c and other repeats. The undegraded OAT3 was displayed as a percentage of the amount of initial OAT3. Values are mean ± S.D. (*n* = 3). * *p* < 0.05.

Our observation that topotecan and GA down-regulated the protein expression of OAT3 (Figure 5 and Figure 6) led us to examine the effects of both compounds on the rate of OAT3 degradation using a biotin-labeling strategy. OAT3-expressing cells were labeled with a membrane impermeable biotinylation reagent, followed by incubation with topotecan (9 h) or GA (6 h) under cell culture conditions. At each time point during the incubation, the biotin-labeled membrane proteins were captured and immunoblotted (IB) with an anti-myc antibody to assess the undegraded OAT3 at the cell surface. The epitope myc was tagged to OAT3 to facilitate its detection. Our data showed that OAT3 degraded at a much faster rate after 3 h treatment of topotecan (Figure 7a,b) and 4 h treatment of GA (Figure 7c,d) when compared to controls.

### 3.6. Effects of Topotecan on Ubc9

**Figure 8:**
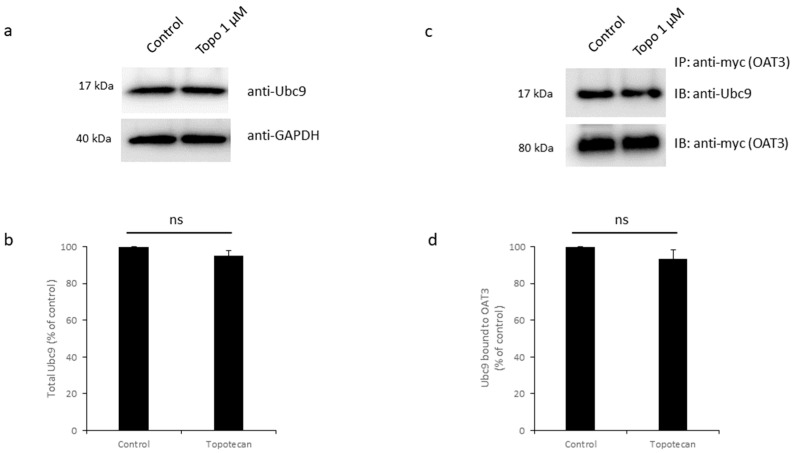
Effects of Topotecan on Ubc9. (**a**) Top Panel: cells expressing OAT3 were treated with topotecan (1 µM, 12 h) and lysed, followed by immunoblotting (IB) with anti-Ubc9 to detect endogenous Ubc9 in the whole-cell lysate. Bottom panel: the same blot was re-probed with anti-GAPDH to assess the total protein marker GAPDH. (**b**) The densitometry of Figure 8a and other repeats. Density values were normalized to GAPDH. Values are mean ± S.D. (*n* = 3). ns, not significant. (**c**) Top Panel: Cells expressing OAT3 were treated with topotecan (1 µM, 12 h). OAT3 was immunoprecipitated (IP) by an anti-myc antibody, followed by immunoblotting (IB) with anti-Ubc9 to assess endogenous Ubc9 that bound to OAT3. Bottom panel: The same blot was re-probed with an anti-myc antibody to assess the amount of OAT3 pulled down. (**d**) The densitometry of Figure 8c and other repeats. Density values were normalized to total OAT3 pulled down. Values are mean ± S.D. (*n* = 3). ns, not significant.

Ubc9 is a crucial SUMO-conjugating enzyme [19,24,43]. However, Bernstock et al. showed that topotecan was unlikely to affect the levels of the conserved E1 (SAE1/SAE2) or E2 (Ubc9) enzymes of the pathway as these proteins were unaffected after exposure to topotecan [29]. Since their study was conducted in a different system from COS-7 cells expressing OAT3, and whether topotecan could affect the binding of Ubc9 with OAT3 was not examined, we, therefore, looked at these possibilities in our system. First, we examined whether topotecan could affect the expression level of endogenous Ubc9. OAT3-expressing cells were treated with topotecan, followed by immunoblotting to detect the expression of Ubc9. Our results (Figure 8a,b) showed that topotecan treatment did not cause a significant change in the total expression of Ubc9. Next, we examined the possibility that topotecan may affect the interaction between Ubc9 and OAT3. OAT3-expressing cells were treated with or without topotecan. OAT3 in treated cells was immunoprecipitated, followed by immunoblotting with an anti-Ubc9 antibody to detect the Ubc9 that was directly bound to OAT3. Our results (Figure 8c,d) showed that topotecan treatment did not markedly alter the interaction between Ubc9 and OAT3.

### 3.7. Effects of Topotecan on SENP2

**Figure 9:**
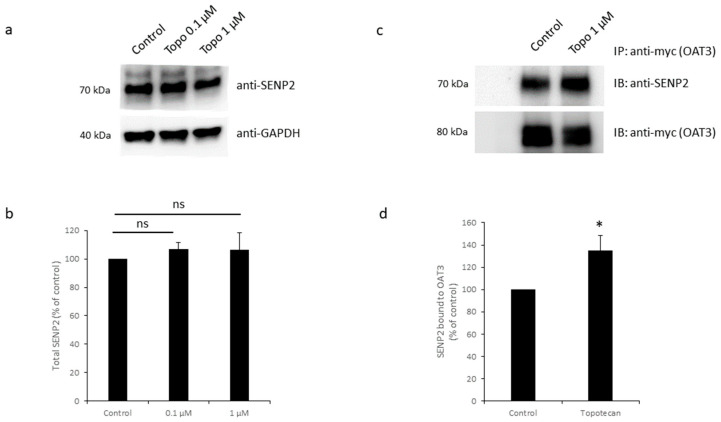
Effects of Topotecan on SENP2. (**a**) Top Panel: cells expressing OAT3 were treated with topotecan (1 µM, 12 h) and lysed, followed by immunoblotting (IB) with anti-SENP2 to measure endogenous SENP2 in the whole-cell lysate. Bottom panel: The same blot was re-probed with anti-GAPDH to detect the total protein marker GAPDH. (**b**) The densitometry of Figure 9a and other repeats. Density values were normalized to GAPDH. Values are mean ± S.D. (*n* = 3). ns, not significant. (**c**) Top Panel: Cells expressing OAT3 were treated with topotecan (1 µM, 12 h). OAT3 was immunoprecipitated (IP) first and immunoblotted (IB) with anti-SENP2 to detect SENP2 that bound to OAT3. Bottom panel: The same blot was re-probed with an anti-myc antibody to assess the total amount of OAT3 pulled down. (**d**) The densitometry of Figure 9c and other repeats. Density values were normalized to total pulled-down OAT3. Values are mean ± S.D. (*n* = 3). * *p* < 0.05.

SENP2 is one of the deSUMOylation proteases identified in our lab [19,24,44]. Our previous study revealed that SENP2 directly interacts with OAT3 both in vitro and in vivo [44]. And, through interacting with OAT3, SENP2 down-regulated the SUMOylation, protein expression, and transporter activity of OAT3 [44]. Thus, we investigated whether topotecan could affect the expression level of SENP2, thereby altering OAT3 SUMOylation. OAT3-expressing cells were treated with topotecan, followed by immunoblotting with an anti-SENP2 antibody to detect the expression of endogenous SENP2. As shown in Figure 9a,b, no meaningful changes in the total expression of SENP2 were observed after topotecan treatment. Next, we explored whether topotecan could affect the direct interaction between SENP2 and OAT3. OAT3 was immunoprecipitated (IP) after topotecan treatment, followed by immunoblotting with an anti-SENP2 antibody to detect the endogenous SENP2 that directly bound to OAT3. Our data (Figure 9c,d) showed that topotecan did enhance the direct interaction between SENP2 and OAT3.

## 4. Discussion

OAT3 plays important roles in renal physiology, renal injury/disease, and the therapeutic efficacy of numerous drugs. Besides transporting numerous endogenous substrates, OAT3 acts as the first step in the active renal elimination of many anionic medicines and their metabolites, including non-steroidal anti-inflammatory drugs, penicillin, and antiviral drugs. As a result, OAT3 is one of the crucial drug transporters required by the FDA to be studied during drug development [1,2,3,6,10].

Our lab previously showed that OATs can be regulated by SUMOylation, a post-translational modification, and that an enhanced SUMOylation of OATs resulted in an enhanced OAT expression and transport activity [13,15,16]. The SUMOylation process requires a network of SUMOylation enzymes: E1, E2, and E3. Firstly, free SUMO molecules are activated by the heterodimer of SUMO-activating enzyme E1 (SAE1/SAE2), which transfers the activated SUMO molecules to a specific SUMO-conjugating enzyme E2 called ubiquitin-conjugating enzyme 9 (Ubc9). A thioester bond is then formed between the activated SUMO molecule and Ubc9 in the second step. Ubc9 usually works together with a SUMO-ligating enzyme E3, which catalyzes the conjugation of SUMO to a target protein. In this last step, the SUMO molecule is covalently conjugated to a lysine residue of the target protein [19,24,45]. Compared to the limited numbers of discovered E1 and E2, multiple proteins have been revealed to have SUMO-ligating activity (E3), such as RAN binding protein 2 and protein inhibitor of activated STAT [19,24,45]. In our current study, we focused on the effects of topotecan and GA on E1 and E2 SUMOylation enzymes. Identifying the specific E3 enzyme that mediates the effects of topotecan and GA on OAT3 will be an exciting direction for us to explore in the future. Additionally, SUMO modification is a dynamic and reversible process where SUMOylated proteins can be deSUMOylated by SUMO-specific proteases. In humans, the sentrin/SUMO-specific proteases (SENPs) are discovered as the first group of SUMO proteases, currently consisting of six members with various target specificity [24,46]. These proteases vary in protein size and sequence, but all share a conserved catalytic domain at the C-terminal. Recent studies have also discovered novel SUMO proteases such as deSUMOylating isopeptidase 1 and ubiquitin-specific protease-like 1, which interestingly have low similarity in amino acid sequence with the classic SENP proteins [19,24,45]. These enzymes can be potential targets for regulating their substrate proteins. Our lab previously discovered that sentrin/SUMO-specific protease 2 is an OAT3-specifc deSUMOylation protease [44].

Recently, GA and topotecan have been reported to affect protein SUMOylation [29,33,36]. However, the effects of both GA and topotecan on OAT3 have not yet been studied. In the current study, we investigated the roles of GA and topotecan in OAT3 SUMOylation, protein expression, and function. We showed that the treatment of OAT3-expressing cells with GA or topotecan resulted in a decrease in OAT3 SUMOylation (Figure 1), which correlated well with a decreased OAT3 protein expression (Figure 5 and Figure 6) and transport activity (Figure 2 and Figure 3). Further mechanistic study revealed that a GA- and topotecan-induced decrease in OAT3 expression is a result of an accelerated rate of OAT3 degradation (Figure 7).

GA exerts its action by directly binding to the E1 enzyme SAE1/2 heterodimer and therefore inhibiting the SUMO activation process [33]. However, the exact site in the SUMOylation/deSUMOylation network where topotecan acts on is not yet known. Therefore, we focused on identifying the exact site in the SUMOylation/deSUMOylation network where topotecan acts on. Our previous work showed that Ubc9 and SENP2 are critical players in the SUMOylation/deSUMOylation process of OAT3: by directly interacting with OAT3, Ubc9 promotes OAT3 SUMOylation, whereas SENP2 promotes OAT3 deSUMOylation [13,24,44,45]. Figure 8 showed that topotecan treatment did not affect Ubc9 protein expression nor its direct interaction with OAT3. Interestingly, for SENP2, although topotecan did not alter its protein expression, the amount of SENP2 directly bound to OAT3 was notably higher with topotecan treatment (Figure 9). This increased binding of SENP2 to OAT3 can lead to more SUMO deconjugation from OAT3 and contribute to the drastically reduced SUMOylation levels of OAT3 we observed in Figure 1.

Protein SUMOylation plays essential roles in various cellular processes, such as protein trafficking and degradation, transcription regulation, cellular response to stress, cell apoptosis, and cell cycle progression [19,23,24,45,47]. The abnormal SUMOylation has been associated with numerous human diseases, including neurodegenerative diseases, diabetes, cardiovascular diseases, and cancer [19,24,45,48,49]. Therefore, targeting components of the SUMOylation network could be a promising treatment strategy for such diseases [45,47,49,50]. The natural products found to inhibit SAE1/2 are GA, anacardic acid, kerriamycin B, and tannic acid [45,51,52]. Most importantly, a mechanistically designed drug candidate (TAK-981 developed by Takeda) targeting SAE1/2 is under clinical trials for treating multiple blood cancers and solid tumors [53,54,55]. This is the first drug candidate targeting the SUMO network to enter the clinical stage. IV administration of TAK-981 drastically reduced the levels of SUMO-conjugated proteins within 24 h in colorectal and lymphoma xenograft tumors in mice. After 21-day treatment under the same dose, TAK-981 significantly shrank the tumor sizes in both xenograft models [53,54,55]. Natural product screening has also discovered spectomycin B1, chaetochromin A, and viomellein as potent Ubc9 inhibitors, which block the formation of the SUMO-Ubc9 conjugate [45,52,56]. In addition, several natural products showed promising inhibitory effects for the SENPs: triptolide, Momordin Ic, and ursolic acid [45,57,58,59,60]. The diverse array of small-molecule inhibitors targeting the SUMO pathway requires continuous investigations to elucidate their unique mechanisms and potential clinical applications.

Protein SUMOylation often takes place at a consensus motif, Ψ-K-X-D/E (Ψ is a hydrophobic amino acid, K is the lysine residue for SUMO conjugation, X is any amino acid, D is an aspartic acid, and E is a glutamic acid) [19,24,61,62]. We used an online software called SUMOplot™ (https://www.abcepta.com/sumoplot, accessed on 10 July 2023) (Abcepta, San Diego, CA, USA) to predict the potential SUMOylation sites of OAT3, and several potential SUMOylation sites on OAT3 with high possibility were identified based on the OAT3 amino acid sequence. However, certain SUMOylation sites can actually occur outside the consensus motif [2,24,61,62]. Thus, direct experimental approaches are required to confirm the actual SUMOylation sites of OAT3, such as site-directed mutagenesis followed by immunodetection and mass spectrometry analysis. Discovering the actual SUMOylation sites of OAT3 would be an interesting direction for our future investigations.

## 5. Conclusions

**Figure 10:**
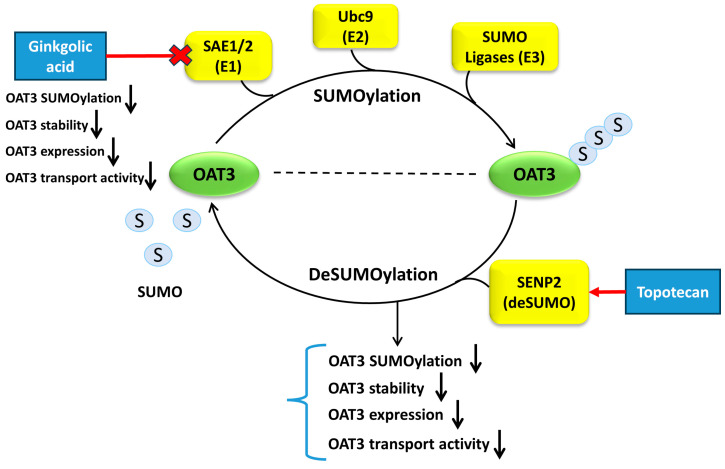
Effects of Topotecan and GA on the SUMOylation, Protein Expression, and Transport Activity of OAT3.

This study uncovered the novel role of topotecan and GA in inhibiting OAT3 protein expression, transport activity, and stability by suppressing the SUMOylation of OAT3 (Figure 10). During comorbidity therapies, the use of topotecan or *Ginkgo biloba* extract could potentially decrease the transport activity of OAT3 in the kidneys, which will in turn affect the therapeutic efficacy and toxicity of many other drugs that are substrates of the transporter. Further investigations in in vivo models would be of great value to confirm these findings and assess their implications for drug therapies and drug–drug interactions.

## Data Availability

The original contributions presented in the study are included in the article; further inquiries can be directed to the corresponding author.
